# Increased ARPP-19 Expression Is Associated with Hepatocellular Carcinoma

**DOI:** 10.3390/ijms16010178

**Published:** 2014-12-24

**Authors:** Haiyan Song, Jielu Pan, Yang Liu, Hongzhu Wen, Lei Wang, Jiefeng Cui, Yinkun Liu, Bing Hu, Zemin Yao, Guang Ji

**Affiliations:** 1 Institute of Digestive Diseases, Longhua Hospital, Shanghai University of Traditional Chinese Medicine, Shanghai 200032, China; E-Mails: songhy@126.com (H.S.); yearlca@126.com (J.P.); liuyang8753com@163.com (Y.L.); ellawen928@yahoo.com (H.W.); et1978@126.com (L.W.); 2 Liver Cancer Institute of Zhongshan Hospital, Fudan University, Shanghai 200032, China; E-Mails: jiefengcui@hotmail.com (J.C.); ykliu@zshospital.com (Y.L.); 3 Department of Oncology and Institute of Traditional Chinese Medicine in Oncology, Longhua Hospital, Shanghai University of Traditional Chinese Medicine, Shanghai 200032, China; E-Mail: beearhu@hotmail.com; 4 Department of Biochemistry, Microbiology and Immunology, Ottawa Institute of Systems Biology, University of Ottawa, Ottawa, ON K1H 8M5, Canada; E-Mail: zyao@uottawa.ca

**Keywords:** hepatocellular carcinoma, ARPP-19, cell proliferation, cell cycle

## Abstract

The cAMP-regulated phosphoprotein 19 (ARPP-19) plays a key role in cell mitotic G2/M transition. Expression of ARPP-19 was increased in human hepatocellular carcinoma (HCC) compared to adjacent non-tumorous liver tissues in 36 paired liver samples, and the level of ARPP-19 in HCC tissues was positively correlated with the tumor size. To determine the interrelationship between ARPP-19 expression and HCC, we silenced ARPP-19 expression in the human hepatocarcinoma HepG2 and SMMC-7721 cells using lentivirus encoding ARPP-19 siRNA. HepG2 and SMMC-7721 cells with ARPP-19 knockdown displayed lowered cell growth rate, retarded colony formation and increased arrest at the G2/M phase transition. Silencing ARPP-19 in HCC cells resulted in decreased protein levels of phospho-(Ser) CDKs substrates and increased levels of inactivated cyclin division cycle 2 (Cdc2). Therefore, ARPP-19 may play a role in HCC pathogenesis through regulating cell proliferation.

## 1. Introduction

Hepatocellular carcinoma (HCC) remains the sixth most common malignancy and third most frequent cause of cancer-related death worldwide [1]. Patients diagnosed with HCC usually have a poor prognosis because of its aggressive nature. Surgical resection or local ablation therapy is effective only at early stages, and approximately 70% of these patients develop recurrent tumors within five years [2,3]. Currently, no effective treatment is available for HCC patients at advanced stage, and molecular target therapy has been considered as a potential intervention for HCC patients. However, cellular or molecular mechanisms underlying HCC development are still poorly defined, and thus, therapeutic strategies for prevention or treatment of advanced-stage HCC are largely lacking [4]. To date, the only proven effective target medicine for HCC, Sorafenib, could prolong the median overall survival for merely <3 months in phase 3 trial [5]. Therefore, there is still a need in identifying targetable cellular and molecular alterations in HCC patients.

ARPP-19 (cAMP-regulated phosphoprotein 19), a member of the alpha-endosulfine (ENSA) family, is discovered in mammalian brain as* in vitro* substrates for protein kinase A [6]. This molecule is ubiquitously expressed and is highly homologous to alpha-endosulfine. Database searches have identified ARPP-19-related proteins in *Drosophila melanogaster*, *Caenorhabditis elegans*, *Schistosoma mansoni* and yeast genomes [7,8]. In the neuronal system, ARPP-19 plays a role in promoting axon growth and synaptic plasticity by providing a link between nerve growth factor signaling and post-transcriptional control of neuronal gene expression [9]. A decreased ARPP-19 level may contribute to the pathomechanisms of Down syndrome and Alzheimer’s disease [10].

**Figure 1:**
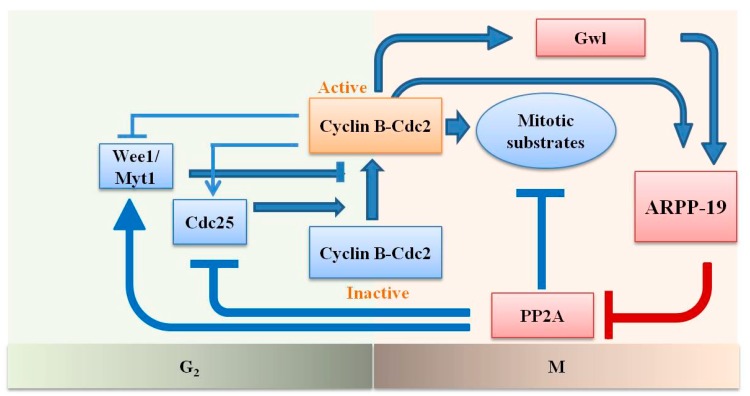
Sketch of the activation of cyclin B-Cdc2 through an autoregulatory loop and the role of ARPP-19 during mitotic entry. The small starter amount of active cyclin B-Cdc2 inactivates Wee1/Myt1 and activates Cdc25 to further activate a larger population of cyclin B-Cdc2. PP2A is the phosphatase that antagonizes these effects of cyclin B-Cdc2. Full activation of Cdc2 cannot take place unless PP2A is inhibited. Gwl is activated by the threshold amount of Cdc2 activity. Gwl, in turn, activates ARPP-19, which binds and inhibits PP2A. ARPP-19 is reported to be also activated directly by Cdc2. The pathway in red denotes the role of ARPP-19.

Recent studies have demonstrated a novel function of ARPP-19 in cell mitosis [11,12]. Cyclin B-Cdc2 (cell division cycle 2, also known as cyclin-dependent kinase 1, Cdk1) is a universal regulator of the M phase of the cell cycle. ARPP-19 was identified to act as a substrate of greatwall (Gwl), which is a kinase with a key function in the activation and maintenance of cyclin B-Cdc2 activity during the G2/M transition [11,13]. ARPP-19, in turn, binds and suppresses the activity of phosphatase 2A (PP2A) with the subunit B55, which inhibits Wee1/Myt1 kinase, which phosphorylates Cdc2 for inhibition and Cdc25 phosphatase, which dephosphorylates the Wee1/Myt1 sites for activation. Gwl could be activated by a starter amount of activated Cdc2. In addition, a recent study reported that cyclin B-Cdc2 directly phosphorylates ARPP-19 on a different conserved site to inhibit PP2A. Thus, Gwl-ARPP-19-PP2A is the core element of the autoregulatory loop of cyclin B-Cdc2 (Figure 1) [14,15,16].

To date, there is no report of the relationship of ARPP-19 and tumorigenesis. The current study thus assessed the level of ARPP-19 expression in human HCC and compared it to that in paired adjacent non-tumor liver tissues and examined the potential effect of ARPP-19 on cell proliferation and the cell cycle.

## 2. Results

### 2.1. ARPP-19 Expression in HCC and Corresponding Non-Tumorous Liver Tissues

**Figure 2:**
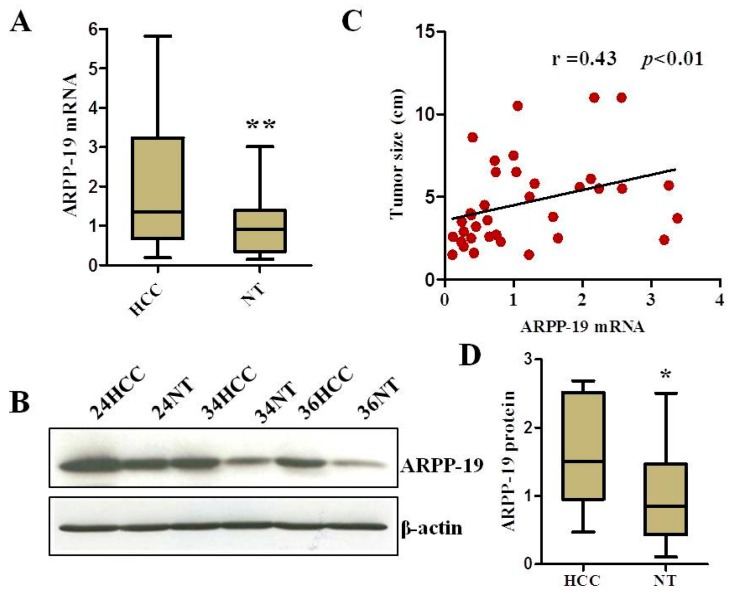
Expression of ARPP-19 in HCC. (**A**) ARPP-19 expression at the mRNA level in 36 pairs of HCC and corresponding non-tumorous liver tissues (NT) was examined by qRT-PCR, with the combination of TBP and SRSF as the internal reference genes (*n* = 36, ** *p* < 0.01); (**B**) Representative western blots of ARPP-19 protein in HCC and non-tumorous liver tissues (NT) samples. β-actin served as a loading control; (**C**) Correlation of the ARPP-19 expression level with the maximal tumor size in HCC (*n* = 36, *r* = 0.43, *p* < 0.01); (**D**) Quantification of (**B**) (*n* = 10, * *p* < 0.05).

The expression of ARPP-19 was compared between 36 pairs of HCC and the corresponding non-tumorous liver tissue (NT) of the same patient. The results of qRT-PCR (Figure 2A) demonstrated that the ARPP-19 mRNA level was significantly increased in HCC, as compared to that in the relative normal liver tissue (*p* < 0.01). Likewise, the ARPP-19 protein level was also increased in HCC (Figure 2B,D).

We further examined possible associations between the levels of ARPP-19 mRNA and the clinicopathologic parameters of the HCC patients, including age, gender, etiology, maximal tumor size, histologic grade, presence of cirrhosis and serum alpha-fetoprotein (AFP) concentration. A significant positive association was observed between the ARPP-19 mRNA level and tumor diameter size (Spearman *r* = 0.43, *p* < 0.01; Figure 2C and Table 1), but not with other clinicopathologic parameters. Correlation analysis with the proliferation rate or patient survival was not performed, because of incomplete data.

**Table 1:** Analysis of the correlation between the expression of ARPP-19 in HCC and the clinicopathological characteristics of HCC patients.

Variable	Category	Number	ARPP-19 Median (Range)	*p*-Value
Age (years)	≤50	16	0.77 (0.12, 2.2)	0.23
	>50	30	0.91 (0.11, 3.4)	
Gender	Male	31	1.0 (0.24, 3.4)	0.08
	Female	5	0.28 (0.11, 2.1)	
Etiology	HBV	30	0.74 (0.11, 3.3)	0.17
	HCV	2	0.99 (0.74, 1.2)	
	Unknown	4	2.1 (0.28, 3.4)	
Histologic grade	I–II	17	0.58 (0.11, 2.0)	0.06
	III–IV	19	1.0 (0.12, 3.4)	
Tumor size	≤5 cm	22	0.52 (0.11, 3.4)	0.009
	>5 cm	14	1.6 (0.41, 3.3)	
Liver Cirrhosis	Absent	5	0.74 (0.25, 3.3)	0.91
	Present	31	0.82 (0.11, 3.4)	
Serum AFP	≤20 ng/mL	16	0.7 (0.11, 3.3)	0.18
	>20 ng/mL	20	1.0 (0.12, 3.4)	

AFP, alpha-fetoprotein; Spearman’s rank correlation was performed between the expression of ARPP-19 and each of the clinical variables.

### 2.2. Silencing ARPP-19 Expression in Hepatocarcinoma Cells

**Figure 3:**
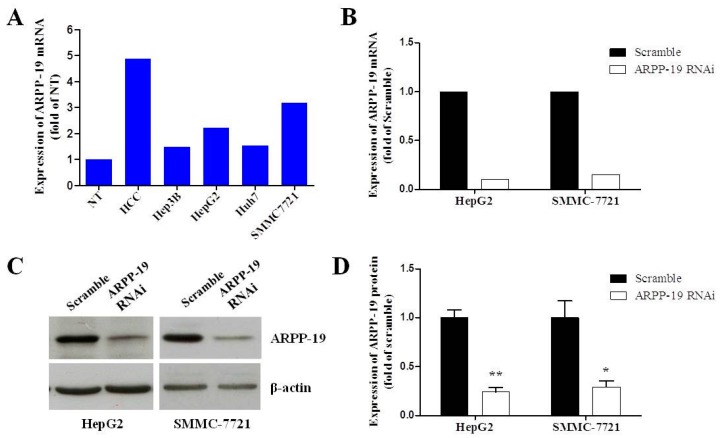
Down-regulating the expression of ARPP-19 in hepatocarcinoma cells. (**A**) The expression level of ARPP-19 in four strains of hepatocarcinoma cells and in the pool of ten pairs of HCC and the non-tumorous liver tissues (NT) was determined by qRT-PCR, with β-actin as the internal control; (**B**) HepG2 and SMMC-7721 cells were transfected with lentivirus with GV118-GFP vector carrying siRNA against ARPP-19 (ARPP-19-RNAi) or empty vector (scramble). The ARPP-19 mRNA level was measured by qRT-PCR, with β-actin as the internal control; (**C**) The protein level of ARPP-19 in HepG2 and SMMC-7721 cells transfected with ARPP-19-RNAi or scramble was measured by immunoblotting, with β-actin as the loading control; (**D**) The images of western blots of (**C**) were quantified. The data are shown as the mean ± SEM, and significant values are indicated with asterisks (* *p* < 0.05; ** *p* < 0.01,* versus* scramble).

In order to study the role of ARPP-19 in HCC development* in vitro*, we first measured its expression in hepatocarcinoma cell lines. Figure 1A shows that more ARPP-19 was expressed in four strains of hepatocarcinoma cells than non-tumorous liver tissues, especially in HepG2 and SMMC7721 cells. Additionally, as HepG2 is well differentiated and SMMC7721 is more malignant with low differentiation, we chose these two cells for further* in vitro* studies by using RNA interference. We next determined the potential role that ARPP-19 plays in controlling cell growth by silencing ARPP-19 expression in HepG2 and SMMC-7721 cells using lentivirus-encoded ARPP-19-RNAi. Transfection efficiency, as determined using a viral vector encoding green fluorescent protein observed with a fluorescence microscope and compared with phase-contrast images, was nearly 100% after transfection. The ARPP-19 protein concentrations were decreased by 75.6% and 73.5%, respectively, in HepG2 and SMMC-7721 cells 72 h after virus transfection (Figure 3C,D). These results demonstrated that the expression of ARPP-19 was successfully downregulated in HepG2 and SMMC-7721 cells under these conditions.

### 2.3. Down-Expression of ARPP-19 Inhibits Cell Proliferation

**Figure 4:**
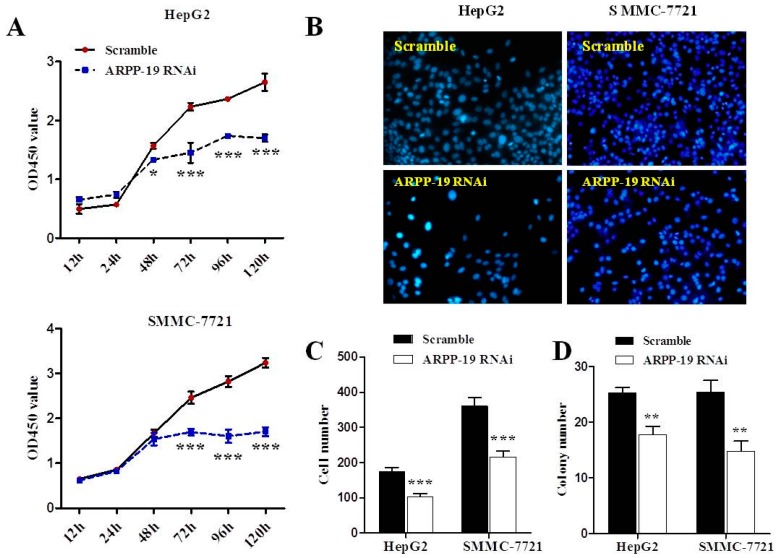
Down-regulated ARPP-19 inhibits the proliferation of hepatocarcinoma cells. (**A**) Cell proliferation was determined using the CCK8 assay. HepG2 or SMMC-7721 cells transfected with lentivirus with siRNA of ARPP-19 (ARPP-19 RNAi) or scramble were seeded into 96-well plates in triplicate. After culturing for 12, 24, 48, 72, 96 or 120 h, the medium was replaced with fresh medium with CCK-8 reagent, followed by 4 h of incubation. The OD value at 450 nm was measured; (**B**) HepG2 or SMMC-7721 cells were cultured in six-well plates for 72 h before being fixed and stained with DAPI. Ten separate fields were imaged under an inverted fluorescence microscope with a magnification of 200×; (**C**) The cell number in (**B**) was counted and analyzed; (**D**) Cells were cultured on soft agar in six-well plates. After 10 days, the formed cell clones containing over 50 cells were counted with the aid of a microscope. All data are shown as the mean ± SEM, and significant values are indicated with asterisks (* *p* < 0.05; ** *p* < 0.01; *** *p* < 0.001,* versus* scramble).

The effect of ARPP-19 depletion on HepG2 and SMMC-7721 cell proliferation was determined by monitoring the cell growth rate. Results of the CCK-8 assay revealed that silencing ARPP-19 began to show retarded cell proliferation at 48 h (*p* < 0.05) in HepG2 cells and had further effect at 72, 96 and 120 h in both cell lines (*p* < 0.001) (Figure 4A). Significantly reduced cell numbers in ARPP-19-depleted HepG2 and SMMC-7721 cells were also observed by cell counting (*p* < 0.001; Figure 4B,C). The colony formation assay showed that ARPP-19-depleted cells grew slowly and formed a few small colonies compared with control cells during a 10-day period (*p* < 0.01; Figure 4D). These results suggest that lowered expression of ARPP-19 results in a reduced rate of hepatocarcinoma cell growth and proliferation.

### 2.4. Down-Expression of ARPP-19 Affects Cell Cycle at G2/M Phase

**Figure 5:**
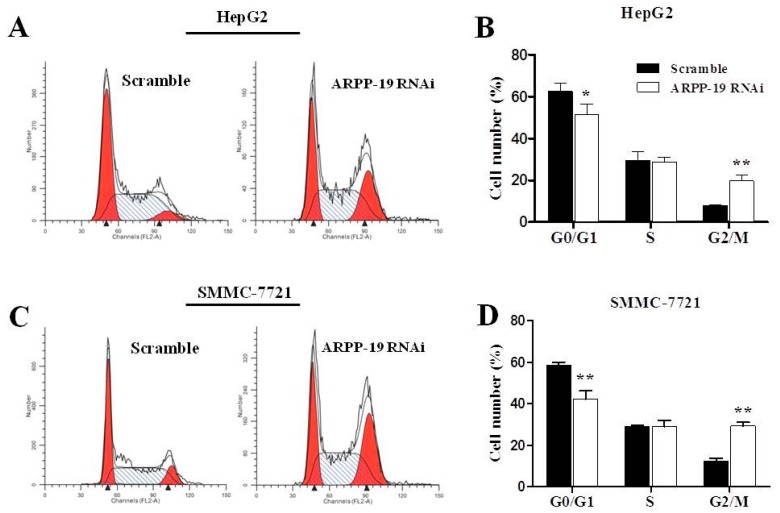
Down-regulated ARPP-19 leads to G2/M cell cycle arrest in hepatocarcinoma cells. (**A**) FACS analysis of HepG2 cells. Cells were cultured for 72 h. All floating and attached cells were collected, stained with propidium iodide and subjected to FACS analysis to examine their cell cycle distributions; (**B**) The percentage of HepG2 cells in each phase of the cell cycle; (**C**) FACS analysis of SMMC-7721 cells; (**D**) The percentage of SMMC-7721 cells in each phase of the cell cycle. All data are shown as the mean ± SEM of three independent experiments, and significant values are indicated with asterisks (* *p* < 0.05; ** *p* < 0.01,* versus* scramble).

Cell cycle deregulation is a common feature of human cancer. Cell cycle arrest plays a critical role in the inhibition of cell proliferation. We subsequently investigated the effect of ARPP-19 silencing on cell cycle distributions. Flow cytometry of ARPP-19 RNAi cells showed a significant increase (>2-fold) in the number of both HepG2 and SMMC-7721 cells bearing G2/M DNA content as compared with scramble (*p* < 0.01; Figure 5A–D), suggesting that knocking down of the endogenous ARPP-19 leads to G2/M cell cycle arrest. In addition, we also observed less cells entering the G0/G1 phase in ARPP-19 RNAi cells.

**Figure 6:**
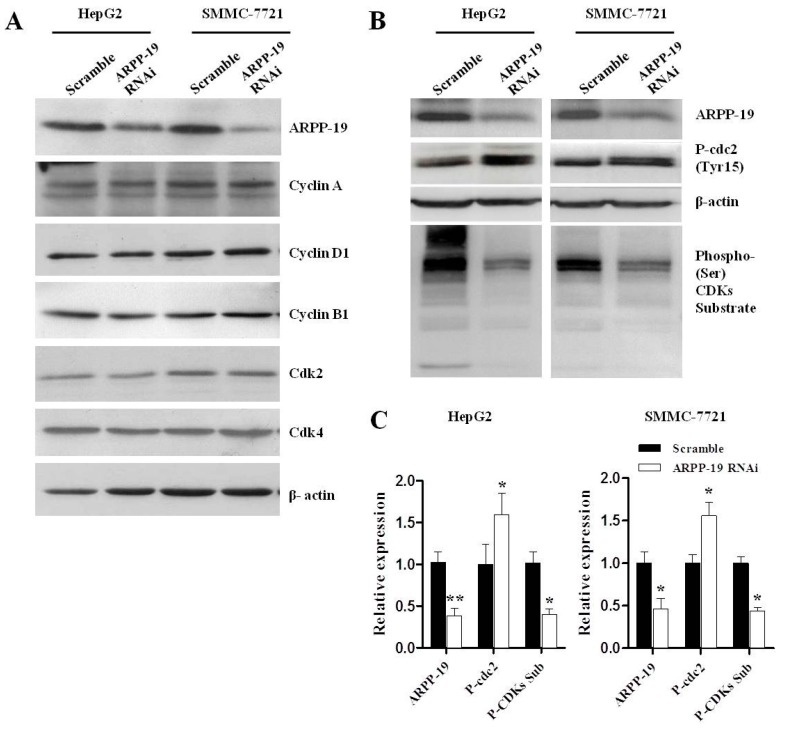
Down-regulated ARPP-19 resulted in decreased phosphorylated mitotic substrates. (**A**) The protein levels of ARPP-19, cyclin A, cyclin D1, cyclin B1, Cdk2 and Cdk4 in HepG2 or SMMC-7721 cells were analyzed by western blot, with β-actin serving as an internal reference; (**B**) HepG2 or SMMC-7721 cells were synchronized at the M phase by thymidine and released into nocodazole for 16 h. The protein levels of ARPP-19, phospho-cdc2 (Tyr15) and the phosphorylation of the different cyclin B-Cdc2 substrates (phospho-(Ser) CDKs substrate) were analyzed by western blot, with β-actin serving as an internal reference; (**C**) Quantification of data in (**B**). All data are shown as the mean ± SEM of three independent experiments, and significant values are indicated with asterisks (* *p* < 0.05; ** *p* < 0.01,* versus* scramble).

The mammalian cell cycle is promoted by a subfamily of cyclin-dependent kinases (Cdks), which, when complexed with specific regulatory proteins, called cyclins, drive the cell forward through the cell cycle [17]. We compared the expression of several cyclins and Cdks between cells with ARPP-19 RNAi or scramble, including cyclin A, cyclin D1, Cdk2, Cdk4 and cyclin B1, which may contribute to modulating the G0/G1 or G2/M phase. However, no obvious difference was found (Figure 6A).

Cell division, including chromatin condensation, spindle formation and sister chromatids separation, requires the regulation of mitotic substrates, which are phosphorylated and activated by Cdc2 and control the G2/M transition directly. We thus determined the effect of ARPP-19 silencing on the level of phosphorylation of Cdc2 substrates in the synchronized mitotic cells by using an antibody recognizing the phospho-serine Cdk consensus motif. As shown in Figure 6B,C, downregulation of ARPP-19 significantly attenuated phosphorylation of mitotic substrates in HepG2 (*p* < 0.01) and SMMC-7721 cells (*p* < 0.05). A significantly elevated level of inactivated Cdc2 (phospho-cdc2 (Tyr15)) was also observed in ARPP-19-depleted hepatocarcinoma cells (*p* < 0.05), indicating that down-regulation of ARPP-19 attenuated the activation of Cdc2.

## 3. Discussion

Chronic viral infection with HBV or HCV appears to be the most significant cause of HCC. In Asia, especially, HBV is the prominent underlying etiology of HCC [1]. This is consistent with our clinical HCC specimen, of which 84.6% were HBsAg (+) and 3.8% HCV. In our ARPP-19 qPCR assay, the housekeeping genes, TATA-binding protein (TBP) and arginine/serine-rich splicing factor 4 (SFRS4 or SRSF4), were used as internal control genes, because TBP and SRSF4 were considered as the most reliable reference genes for q-PCR normalization in HBV- or HCV-related HCC specimens, respectively [18,19]. Our results showed higher expression of ARPP-19 in HCC compared to that in the paired non-tumorous liver tissues, and the level of ARPP-19 was positively correlated with the tumor size. In addition,* in vitro* experiments demonstrated that ARPP-19 knockdown suppressed the proliferation and colony formation of HepG2 and SMMC-7721 cells. This is consistent with the report that silencing ARPP-19 reduced the number of mitotic cells by 50% in the human cervical cancer HeLa cells [11].

Previous studies have shown that ARPP-19 was highly expressed in embryonic tissues, and its expression decreased progressively during development. A high level of expression of ARPP-19 was found in malignant cell lines, which is consistent with our result that a higher level of ARPP-19 was expressed in hepatocarcinoma cells than the corresponding normal liver tissues, suggesting that ARPP-19 over-expression may be associated with cell malignancy [8]. HCC develops from chronic liver disease, during which continuous inflammation occasionally damages DNA in the hepatocytes of the regenerating liver, thereby increasing the chances of gene alteration related to carcinogenesis. These alterations include constitutively upregulated expression of factors involved in “stemness” with the characteristic of unlimited proliferation [20]. Therefore, our observation is consistent with the notion that ARPP-19 over-expression contributes to HCC tumorigenesis.

The cell cycle is a critical regulator of the processes of cell proliferation and growth. It is typically divided into four phases. The periods associated with DNA synthesis (S phase) and mitosis (M phase) are separated by gaps of varying length, called G1 and G2. In this study, the G2/M phase was arrested after ARPP-19 was down-regulated. We also observed a reduced ratio of cells in the G0/G1 phase, which may be due to less cells entering the new cycle. Specific cyclin-Cdks promote the progression in a timely manner. Cyclin D interacts with Cdk2, Cdk 4 and Cdk 6 and drives a cell’s progression through G1. The association of cyclin E with Cdk2 is active at the G1/S transition and directs entry into the S phase. S phase progression is directed by the cyclin A–Cdk2 complex, and the complex of cyclin A with Cdc2 (Cdk1) is important in G2. Cyclin B–Cdc2 is necessary for mitosis to occur [17]. We analyzed the level of cyclin A, cyclin D1, Cdk2, Cdk4 and cyclin B1, without finding the impact of ARPP-19 absence on these regulating molecules.

Historically, mitotic entry and exit was thought to be equivalent to cyclin B–Cdc2 activation and inactivation. However, recent studies have indicated that initiation and maintenance of mitosis require not only the activation of protein kinase cyclin B–Cdc2, but also the inhibition of the “antimitotic” protein, phosphatase 2A (PP2A), which, respectively, phosphorylate and dephosphorylate mitotic substrates [21]. The protein kinase, Gwl, plays a crucial role in inactivating PP2A mediated by ARPP-19 [11,13]. Depletion of either ARPP-19 or Gwl by siRNA arrests *Xenopus* egg, or human Hela cells in G2, or renders cells unable to activate the spindle assembly checkpoint and subsequently exit from mitosis [11,22]. Microtubule associated serine/threonine kinase-like protein (MASTL) is the functional human ortholog of Gwl. Depletion of MASTL delays the G2-to-M phase transition, is linked to a prolonged mitosis and sensitizes cells to mitotic death [23]. The present study showed similar results: down-regulation of ARPP-19 resulted in decreased phosphorylated mitotic substrates, which arrested more than two-fold the HepG2 or SMMC-7721 cells at G2/M phase. PP2A could inhibit the activation of Cdc2 by dephosphorylating its key regulators, as well as antagonizing Cdc2’s own phosphorylation of downstream targets [24,25]. Depletion of ARPP-19 increased the activity of PP2A and subsequently failed to dephosphorylate Cdc2 to activate this kinase [11,22]. On the other hand, Gwl-ARPP19 can be also activated directly by activated Cdc2 to inhibit PP2A. Consistently, we also observed higher expression of phospho-cdc2 (Tyr15) in HepG2 cells with ARPP-19 knockdown* versus* scramble. Taken together, these results provide evidence to support that ARPP-19 promotes cell mitosis through the Gwl-ARPP-19-PP2A pathway in human cells, which may also affect the activation of Cdc2 for mitotic entry.

Whether or not ARPP-19 plays a direct role in human diseases is currently unknown. However, previous studies have implicated some of the ARPP-19 regulators or effectors in malignancy. For instance, PP2A, the downstream molecule of ARPP-19, has been reported to function as a tumor suppressor [26]. Phosphorylation of ARPP-19 by cAMP in striatal cells was increased by neurotransmitters VIP (vasoactive intestinal peptide) and dopamine [27]. The effect of VIP on ARPP-19 phosphorylation was also observed in reaggregate striatal cultures [8]. VIP has been shown to play a role in the pathogenesis of breast and prostate cancer [28]. Dopamine receptor (DR) antagonist was found to reduce the incidence of rectum, colon and prostate cancer [29]. DRs are differentially expressed on neoplastic stem cells, suggesting that the dopamine-DR signaling pathway may be considered as a druggable target in cancer [29].

In summary, the current study demonstrates, for the first time, that over-expression of ARPP-19 occurred in HCC, and down-regulation of ARPP-19 expression in hepatocarcinoma cells resulted in attenuated cell growth and proliferation, which may be due to the role of ARPP-19 in the regulation of the cell cycle. Although the number of HCC patients recruited in the current study was too small to conclude definitively that ARPP-19 could serve as a biomarker or a predictor of outcome, we postulate that ARPP-19 may be a potential target for HCC treatment. In addition, other limitations exist in the current study, for example, lacking a group with over-expression of ARPP-19 for study and no* in vivo* animal experiments to prove the results. Further study of ARPP-19 with a large cohort of HCC patients and exploration of the defined signal pathway are required.

## 4. Materials and Methods

### 4.1. Tissue Specimen and Cell Lines

Thirty-six paired HCC tissues and the adjacent non-malignant liver tissues were collected for study from the patients who underwent surgery at the First Affiliated Hospital of Guangxi Medical University (Nanning, China) with informed consent. The patients included 5 women (13.9%) and 31 men (86.1%), with ages ranging from 31 to 83 years. The underlying liver diseases of the HCC were: hepatitis B virus (HBV) infection (30 cases, 83.3%), hepatitis C virus (HCV) infection (2 cases, 5.6%) and unknown aetiologies (4 cases, 11.1%). The characteristics of the involved cases are listed in Table 1. The study was approved by the human ethics committee of Shanghai University of Traditional Chinese Medicine (Identification number: 2012-353; Date: 30 December 2012). The samples were immediately snap-frozen in liquid nitrogen and stored at −80 °C until further processing.

Hep3B, HepG2, Huh7 and SMMC-7721 cells were purchased from the Cell Biology Institute of Chinese Academy of Science, Shanghai, China. HepG2, Huh7 and SMMC-7721 cells were cultured in DMEM (Gibco BRL, Rockville, MD, USA), and Hep3B cells were cultured in MEM (Gibco BRL, Rockville, MD, USA), supplemented with 10% fetal bovine serum, penicillin (100 U/mL) and streptomycin (100 μg/mL) (Biowest, Nuaillé, France), at 37 °C in a humid incubator with 5% CO_2_.

### 4.2. RNA Extraction and Quantitative Reverse Transcription-Polymerase Chain Reaction (qRT-PCR)

The tissue specimen ground in liquid nitrogen or the cultured cells were homogenized in TRIzol (Invitrogen, Carlsbad, CA, USA). Five micrograms of RNA were reverse transcribed using GoScript reverse transcription System (Promega, Madison, WI, USA) to get the cDNA template. Quantitative real-time PCR and data analysis were carried out using an AB StepOne Plus Real-time PCR System (Applied Biosystems, Carlsbad, CA, USA). TaqMan gene expression assays (Applied Biosystems) for ARPP19 (Hs01055630_g1), TBP (Hs00427620_m1), ACTB (Hs99999903_m1) and SRSF (Hs00194538_m1), as well as the TaqMan^®^ Universal Master Mix II were used to quantify mRNA expression through the comparative *C*_t_ (^ΔΔ^*C*_t_) experiment. ARPP19 mRNA expression levels of each sample were calculated with the combination of TBP and SRSF as reference genes.

### 4.3. Western Blot Analysis

Liver tissues or cells were homogenized in RIPA buffer with the proteinase inhibitor (complete mini EASY pack, Roche, Basel, Switzerland) and protein concentration was determined through the BCA method. The lysates were resolved on 12% sodium dodecyl sulfate-polyacrylamide by gel electrophoresis (SDS-PAGE) and transferred to a PVDF membrane (Millipore, Billerica, MA, USA). After blocking, membranes were immunoblotted with the antibody against ARPP-19 (Proteintech, Wuhan, China), cyclin A2, cyclin D1, Cdk2, Cdk4, cyclin B1, phospho-cdc2, phospho-(Ser) CDKs substrate (Cell Signaling Technology, Boston, MA, USA) or β-actin (Huaan Biological Technology, Hangzhou, China) as the internal loading control. The primary antibodies were visualized with goat anti-rabbit (CST) peroxidase-conjugated antibody by an enhanced chemiluminescence detection system (Millipore). The images of blots were acquired by the GBOX Chemi XT4 System (Syngene, Cambridge, UK) and quantified by densitometry with GeneTools software (Syngene).

### 4.4. Knockdown of ARPP-19 in Hepatocarcinoma Cells

From three pairs of small interfering RNAs (siRNA) produced by Invitrogen, one pair was proven efficient in downregulating ARPP-19 expression when transferred into HepG2 cells. According to the siRNA sequence against human ARPP-19 (AAGCCTGGAGGTTCAGATTTCTTAA), the ARPP-19-RNAi lentiviral vector was constructed by GeneChem Co., Ltd. (Shanghai, China), using the vector, GV118-GFP. Then, the specific lentivirus was generated and harvested after 293T cells were transfected by the vector and cultured for 48 h. To establish the stable cell line, the ARPP-19-RNAi lentivirus was transfected into HepG2 and SMMC-7721 cells with a multiplicity of infection (MOI) of 80. After 72 h, the transfection efficiency was observed through a fluorescence microscope, and ARPP-19 expression was determined through western blot analysis. Cells transfected by lentivirus with the empty vector were used as scrambles.

### 4.5. Cell Counting

A total of 2 × 10^5^ cells/well were seeded and cultured for 72 h in 6-well plates. Then, the cells were fixed in 4% paraformaldehyde and incubated for 10 min with 4',6-diamidino-2-phenilindole (DAPI) (Sigma Aldrich, St. Louis, MO, USA). Ten separate fields were imaged under an inverted fluorescence microscope (Olympus, Tokyo, Japan) with a magnification of 200×, and the software, ImageJ 1.47 (http://rsbweb.nih.gov/ij/), was used to count the cell number.

### 4.6. Cell Counting Kit-8 Assay for Cell Proliferation

Cell proliferation was determined using the CCK-8 assay (Dojindo, Kumamoto, Japan) following the manufacturer’s protocol. Briefly, 1 × 10^3^ of HepG2 or SMMC-7721 cells in 100 μL media were seeded into each well of a 96-well plate in triplicate. After culturing for 12, 24, 48, 72, 96 or 120 h, the previous medium was replaced with 100 μL of fresh medium and 10 μL of CCK-8 reagent, followed by 4 h of incubation. The optical density (OD) of the cultures was measured at 450 nm using a Synergy H4 Hybrid Multi-Mode microplate reader (BioTeck, Winooski, VT, USA).

### 4.7. Clone Formation Assay

Base layers of 7 mL of DMEM containing 0.5% Difco Bacto agar (BD Bioscience, Franklin Lakes, NJ, USA) were set in each well of 6-well plates before the addition of a second layer with 1 × 10^3^ cells in 1.5 mL of medium containing 0.3% agar. The plates were incubated for 10 days at 37 °C in the incubator to form clones. Colony counts were made with the aid of a microscope. Five fields were selected randomly, and only clones containing over 50 cells were counted to determine the clone-forming ability of the cells.

### 4.8. Cell Cycle Distribution Analysis

About 2 × 10^5^ cells were incubated in the well of 6-well plates. After 72 h, the cells were harvested, fixed with 70% pre-chilled ethanol, treated with 100 µg/mL RNase A solution (Sigma Aldrich) and stained with a 0.5 mg/mL propidium iodide (PI) solution (Sigma Aldrich) for 30 min. The DNA content in the cells was detected using a flow cytometer (BD Bioscience). The data were analyzed using CellQuestPro software (BD Bioscience).

### 4.9. Cell Synchronization

For mitotic synchronization of cells at the M phase, cells were incubated in medium with 2 mM thymidine (Sigma Aldrich) for 18 h, released into fresh medium for 3 h, treated with 100 ng/mL nocodazole (Sigma Aldrich) for 16 h and shaken off. The floating cells were then collected for protein detection.

### 4.10. Statistical Analyses

SPSS 16.0 (SPSS, Inc., Chicago, IL, USA) and GraphPad Prism 5 (GraphPad Prism software, La Jolla, CA, USA) were used for data analysis. The data were expressed as the mean ± standard error of mean (SEM). Statistical analyses were determined using the two-tailed Student’s *t*-test. The chi-square test was used to analyze nonparametric variants. ARPP-19 expression in HCC and the adjacent tissues was compared by Wilcoxon matched pairs test. The correlation of the associations between the expression of ARPP-19 and various clinicopathologic parameters was calculated according to Spearman.* p* < 0.05 was considered statistically significant.
